# The Association Between the Decline of eGFR and a Reduction of Hemoglobin A_1c_ in Type 2 Diabetic Patients

**DOI:** 10.3389/fendo.2021.723720

**Published:** 2022-01-19

**Authors:** Lingwang An, Qiuzhi Yu, Linhui Chen, Hong Tang, Yanjun Liu, Qun Yuan, Yu Ji, Yaujiunn Lee, Juming Lu

**Affiliations:** ^1^ Department of Endocrinology, Beijing Ruijing Diabetes Hospital, Beijing, China; ^2^ Department of Endocrinology, Heilongjiang Ruijing Diabetes Hospital, Haerbin, China; ^3^ Department of Endocrinology, Taiyuan Diabetes Hospital, Taiyuan, China; ^4^ Department of Share-care Center, Chengdu Ruien Diabetes Hospital, Chengdu, China; ^5^ Department of Endocrinology, Lanzhou Ruijing Diabetes Hospital, Lanzhou, China; ^6^ Department of Endocrinology, Beijing Aerospace General Hospital, Beijing, China; ^7^ Lee’s Endocrinology Clinic, Taiwan, China; ^8^ Department of Endocrinology, The General Hospital of the People’s Liberation Army, Beijing, China

**Keywords:** diabetes, type 2, hemoglobin A_1c_ (HbA_1c_), estimated glomerular filtration rate (eGFR), hyperfiltration

## Abstract

**Objective:**

This study aimed to explore the relationship between short-term (≤12 months) changes in the estimated glomerular filtration rate (eGFR) and hemoglobin A_1c_ (HbA_1c_) in patients with type 2 diabetes (T2D).

**Method:**

A total of 2,599 patients with T2D were enrolled if they were registered in the Diabetes Sharecare Information System, were aged 18–75 years, and had 2–3 HbA_1c_ and eGFR measurements within the preceding 12 months. The studied patients were categorized into five groups based on eGFR, i.e., the relatively stable (RS), fast decline (FD), modest decline (MD), modest increase (MI), and fast increase (FI) groups.

**Results:**

The median eGFR changes from baseline were −22.14, −6.44, 0.00, 6.32, and 20.00 ml/min per 1.73 m^2^ for patients in the FD, MD, RS, MI, and FI groups, respectively. Up to 1,153 (44.4%) subjects experienced an eGFR decline of ≥3.5 ml/min per 1.73 m^2^, including 821 (31.6%) FD subjects and 332 (12.8%) MD subjects. A decreased trend was found between the eGFR change and HbA_1c_ decrease category, even after multivariable adjustment. In general, an eGFR FD was frequently found in patients who had an HbA_1c_ reduction of ≥3.00% and a baseline HbA_1c_ ≥8.0%; alternatively, such a result was also observed for a urinary albumin-to-creatinine ratio (UACR) of 30.0–300.0 mg/g, regardless of a diabetes duration of <10.0 or ≥10.0 years, or in patients who had an HbA_1c_ reduction of ≥1.00% accompanied by hyperfiltration.

**Conclusions:**

Some patients with T2D experienced an eGFR FD or MD during the ≤12-month follow-up period. A significant downward trend in eGFR change was demonstrated alongside an HbA_1c_ reduction, independent of UACR stage, diabetes duration, and hyperfiltration. Sustained monitoring and cautious interpretation of the HbA_1c_ and eGFR changes will be needed in clinical practice.

## Introduction

Diabetes has become a major global public health problem. It is an age-related disease, and its prevalence increases with age (1). With the aging of the population, there is an ongoing increase in the number of patients with diabetes in China. The harm caused by diabetes concerns not only the elevation of sugar levels in the bloodstream but also the complications that arise as a result. Diabetic kidney disease (DKD) is one of the most common complications of diabetes (2, 3), which, in turn, is the main cause of chronic kidney disease (CKD) and can eventually lead to end-stage renal disease and death. According to statistics (4), 25%–40% of patients with type 2 diabetes (T2D) will eventually develop diabetic nephropathy. The diagnosis of DKD is typically based on the presence of albuminuria and/or a reduced estimated glomerular filtration rate (eGFR) in the absence of signs or symptoms of other primary causes of kidney damage (5, 6). Sustained attention should be paid to a change in eGFR and urinary albumin-to-creatinine ratio (UACR), which is the standard method for assessing glomerular damage and renal function changes in clinical practice. The eGFR is generally considered an important predictor of overall renal function; it is typically calculated with serum creatinine using the Chronic Kidney Disease Epidemiology Collaboration (CKD-EPI) equation or the simplified Modification of Diet in Renal Disease (MDRD) equation and is reported by laboratories (7, 8). The normal eGFR decline was 1 ml/min per year after the age of 40 (9). The slope of eGFR changes over time varied widely among individuals with type 1 diabetes (T1D), from −72 to −3.0 ml/min per 1.73 m^2^ per year, and was reflected as a very fast, fast, moderate, or slow decline; however, less evidence was found in participants with T2D (10, 11). The eGFR changes indicated the progression of CKD in patients with diabetes. Furthermore, the eGFR equations were less accurate in the diabetic than in the non-diabetic group, regardless of using the CKD-EPI or MDRD equations, and hemoglobin A_1c_ (HbA_1c_) was an independent factor associated with accuracy in eGFR equations (12). The eGFR value after glycemic control will thus be clinically more meaningful.

HbA_1c_ is an important indicator for observing whether or not the condition of diabetes is being well controlled; it reflects the average blood glucose level 2–3 months prior to a medical examination (13) and has been widely used in clinical practice. It is also a good indicator of diabetes diagnosis, efficacy evaluation, the observation of treatment compliance, and prognosis judgment and plays an important role in evaluating the occurrence and development of various diabetes complications. It was shown that HbA_1c_ reduction after a few months was associated with a significant reduction in eGFR, and each +1% HbA_1c_ was associated with +5.3 ml/min per 1.73 m^2^ eGFR using the MDRD equation (14). However, there are few reports on the relationship between changes in HbA_1c_ level and eGFR in T2D patients. Accordingly, we studied the association between short-term changes in eGFR and HbA_1c_ in patients with T2D during a 12-month follow-up period.

## Materials and Methods

### Study Design

This was an observational, multicenter, retrospective study based on medical records included in the Diabetes Sharecare Information System (DSIS) of Ruijing diabetes chain hospitals (RDCHs), five primary care medical institutes located in the cities of Beijing, Taiyuan, Chengdu, Harbin, and Lanzhou. This DSIS was developed for diabetic patients’ registration, follow-up, and preservation of clinical and biochemical measurements. Only patients who provided oral consent for inclusion in the study were allowed to be registered. RDCHs began using DSIS in 2016. All data were aggregated for each person after registration (baseline) and during each follow-up visit. The analysis was based on individuals with a documented T2D onset and clinical visits at the baseline measurement and second/third follow-ups. The Declaration of Helsinki guidelines were followed while conducting this study. The research was approved by the Ethics Committee of Beijing Ruijing Diabetes Hospital. Due to the nature of the study (i.e., retrospective/database), patient consent was not required.

### Study Population

**Figure 1:**
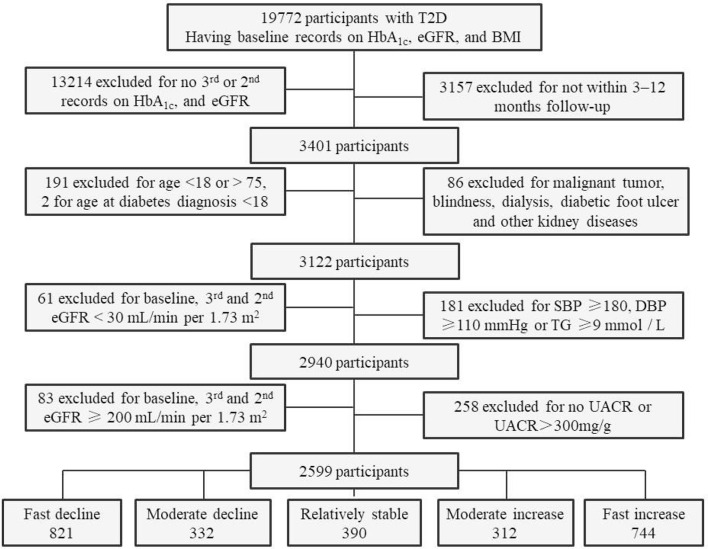
Flowchart and sample size of participants for the final analyses. T2D, type 2 diabetes; eGFR, estimated glomerular filtration rate; BMI, body mass index; SBP, systolic blood pressure; DBP, diastolic blood pressure; TG, triglyceride; UACR, urinary albumin-to-creatinine ratio.

The inclusion criteria for the present study were patients with T2D, aged 18–75 years, age at diabetes diagnosis ≥18 years, patients who had second (if no third) or third records of HbA_1c_ and eGFR values at the 12-month follow-up visits. Patients were excluded if they had a history of T1D, gestational and/or secondary diabetes, a malignant tumor, blindness, dialysis, diabetic foot ulcers, other kidney diseases; no medical records of baseline HbA_1c_, eGFR, or body mass index (BMI) and eGFR values <30.00 or ≥200 ml/min per 1.73 m^2^ at the baseline or follow-up visits; or uncontrolled blood pressure [systolic blood pressure (SBP) ≥180 mmHg, diastolic blood pressure (DBP) ≥110 mmHg], a triglyceride (TG) level ≥9 mmol/L (which typically causes chylous blood) (15), and a UACR ≥300 mg/g. The inclusion period lasted from January 27, 2016, to April 26, 2020. The final cohort comprised 2,599 adults with T2D (**Figure 1**).

### Measures and Category

The HbA_1c_ measurements were tested with high-performance liquid chromatography using HA-8180 (ARKAY, Inc., Kyoto, Japan) and MQ-2000PT (Medconn Diagnostics, Shanghai, China) chromatograph, standardized according to the Diabetes Control and Complications Trial (16). Biochemical parameters, including serum creatinine, low-density lipoprotein cholesterol (LDL-C), and urinary albumin and creatinine were tested by automatic biochemical analyzers using a TBA-120FR (Toshiba, Beijing, China), CS-1200 (DIRUI, Changchun, China) and BS-450 (Mindray, Shenzhen, China) in regular quality control and meeting the local internal quality control standards. Clinical measurements of HbA_1c_ and serum creatinine were obtained from the laboratory database from the time of cohort entry to the second (if no third) record or third follow-up exam. BMI was categorized as not overweight/obese: <24.0 kg/m^2^ and overweight/obese: BMI ≥24.0 kg/m^2^. The target LDL-C was <2.60 mmol/L; target blood pressure (BP) was <130/80 mmHg (16). Albuminuria was categorized as normal (stage A1): UACR <30 mg/g; microalbuminuria (stage A2): UACR was 30–300 mg/g; macroalbuminuria (stage A3): UACR >300 mg/g (6, 17).

The simplified (Chinese) MDRD equation was used to calculate the eGFR as follows: MDRD = 175× serum creatinine (mg/dl) ^−1.234^ × years ^−0.179^ × (0.79, if female) (8). According to changes in serum creatinine or eGFR from the second/third visit compared with the baseline, patients were categorized into five groups, i.e., a relatively stable (RS) control group, eGFR change −3.49 to 3.49 ml/min per 1.73 m^2^; fast decline (FD), where eGFR change <−10.00 ml/min per 1.73 m^2^; a moderate decline (MD) group, where eGFR change was −10.00 to −3.50 ml/min per 1.73 m^2^; a moderate increase (MI) group, where eGFR change was 3.50–10.00 ml/min per 1.73 m^2^; a fast increase (FI) group, where eGFR change was >10.00 ml/min per 1.73 m^2^ (10, 11). The CKD stages were defined as follows: G1, eGFR ≥90.0 ml/min per 1.73 m^2^; G2, eGFR 60.0–89.9 ml/min per 1.73 m^2^; G3, eGFR 30.0–59.9 ml/min per 1.73 m^2^. Stages 4/5 for CKD were not included (5, 18). The hyperfiltration was defined as eGFR >120 ml/min per 1.73 m^2^ or, alternatively, using age-unadjusted eGFR threshold (1) 18–49 years, eGFR >120 ml/min per 1.73 m^2^; (2) 50–59 years, eGFR >110 ml/min per 1.73 m^2^; (3) 60–69 years, eGFR >100 ml/min per 1.73 m^2^; (4) 70–75 years, eGFR >90 ml/min per 1.73 m^2^ (9, 19).

### Medication

Medication information was obtained on the registration date and during follow-up visits. Treatment for hyperglycemia was categorized into two groups, i.e., a non-insulin [lifestyle, oral anti-diabetic (OAD) drugs or glucagon-like peptide-1 receptor agonist (GLP-1RA)] and insulin (insulin only or insulin plus other management) usage. Whether the patients used sodium-glucose cotransporter-2 inhibitors (SGLT-2i), GLP-1RA, and angiotensin-converting enzyme inhibitors (ACEIs)/angiotensin receptor blockers (ARBs) was also categorized.

### Statistical Analysis

Patient characteristics were summarized using mean and standard deviation and median and interquartile range or numbers (percentages). Additionally, 95% confidence intervals were reported. One‐way analysis of variance tests were used for normally distributed continuous variables, Kruskal–Wallis tests were used for non-normally distributed continuous variables, and Pearson’s chi‐square tests were used for categorical variables. Interactions with HbA_1c_ changes were determined. Subgroup analyses were conducted according to baseline HbA_1c_ category (HbA_1c_ <8.0 and ≥8.0%), baseline hyperfiltration (yes or no), baseline BMI category (BMI <24.0 and ≥24.0 kg/m^2^), and diabetes duration (<10.0 and ≥10.0 years). Multivariable-adjusted general linear models (adjusted for age, gender, disease duration, baseline hyperfiltration, BMI and HbA_1c_ category, UACR stage, follow-up insulin, SGLT-2i, ACEI/ARB usage, and BP control) were reported separately. A two-sided *P*-value of <0.05 was considered to be statistically significant. All analyses were performed using the SPSS Statistics 22.0 software.

## Results

### Patient Characteristics

The final analytic cohort comprised 2,599 individuals with T2D, with a median age at entry of 59.7 years, diabetes duration of 8.2 years, and follow-up of 8 months. The proportion of patients that had a history of hypertension, dyslipidemia, diabetic retinopathy (DR), or DKD was 37.9%, 33.5%, 14.7%, and 9.7%, respectively (**Table 1**).

**Table 1:** Demographic and clinical characteristics for the overall study population.

Variables	Overall (n = 2,599)
Age, years	59.7 (53.2–65.3)
≥60 years, n (%)	1,279 (49.2)
Gender (male), n (%)	1,514 (58.3)
Diabetes duration (years)	8.2 (3.7–12.3)
≥10.0, n (%)	1,075 (41.4)
Follow-up (months)	8.0 (6.0–11.0)
Hypertension, n (%)	985 (37.9)
Dyslipidemia, n (%)	871 (33.5)
Retinopathy, n (%)	383 (14.7)
DKD, n (%)	253 (9.7)
Current smoker, n (%)	406 (22.6)
Current drinker, n (%)	396 (21.8)

Data are numbers (%) or medians [interquartile range (IQR)].

BMI, body mass index; eGFR, estimated glomerular filtration rate; UACR, urinary albumin-to-creatinine ratio; DKD, history of diabetic kidney disease.

### Comparisons Among the 5 eGFR Change Groups

The median eGFR changes from baseline were −22.14, −6.44, 0.00, 6.32, and 20.00 ml/min per 1.73 m^2^ for patients in the FD, MD, RS, MI, and FI groups. Up to 1,153 (44.4%) of the study subjects experienced an eGFR decline of ≥3.5 ml/min per 1.73 m^2^, including 821 (31.6%) FD subjects and 332 (12.8%) MD subjects. The baseline eGFR, HbA_1c_, the age-adjusted prevalence of hyperfiltration decreased from group FD to FI, while HbA_1c_ change increased gradually from group FD to RS (*P* < 0.001) (**Table 2**). Baseline and follow-up BMI and BP and LDL-C control medication use, including insulin, SGLT2i, GLP-1RA, and ACEI/ARB, were reported (**Table 3**).

**Table 2:** Characteristics among subjects stratified by eGFR changes.

Variables	FD	MD	RS	MI	FI	*P*
	(n = 821)	(n = 332)	(n = 390)	(n = 312)	(n = 744)	
Baseline						
eGFR (ml/min per 1.73 m^2^)	131.3 ± 30.9	115.7 ± 30.3	108.0 ± 30.9	102.6 ± 31.3	97.1 ± 26.7	<0.001
HbA_1c_ (%)	8.1 (6.8–9.8)	8.0 (6.8–9.5)	7.6 (6.6–9.2)	7.5 (6.5–8.9)	7.6 (6.6–8.9)	<0.001
Hyperfiltration, n (%)	644 (78.4)	194 (58.4)	195 (50.0)	131 (42.0)	260 (34.9)	<0.001
UACR stage						0.001
A1	602 (73.3)	248 (74.7)	307 (78.7)	225 (72.1)	595 (80.0)	
A2	219 (26.7)	84 (25.3)	83 (21.3)	87 (27.9)	149 (20.0)	
Follow-up						
eGFR (ml/min per 1.73 m^2^)	104.3 ± 27.6	109.1 ± 30.3	107.9 ± 31.0	109.2 ± 31.3	121.4 ± 28.6	<0.001
HbA_1c_ (%)	7.1 (6.4–8.2)	7.3 (6.4-8.4)	7.2 (6.5-8.4)	7.0 (6.3-8.2)	7.2 (6.5-8.4)	0.110
eGFR change (ml/min per 1.73 m^2^)	-22.14 (-33.44 to -15.50)	-6.44 (-8.37 to -4.92)	0.00 (-1.88 to 1.62)	6.32 (5.03–8.43)	20.00 (14.39–29.96)	<0.001
HbA_1c_ change (%)	-0.60 (-2.10 to 0.10)	-0.40 (-1.50 to 0.30)	-0.20 (-1.10 to 0.40)	-0.20 (-0.90 to 0.30)	-0.20 (-0.90 to 0.40)	<0.001

Categorical variables were expressed as numbers (%). Continuous variables were expressed as medians [interquartile range (IQR)] for non-normally distributed variables or mean ± standard deviation for normally distributed variables.

eGFR, estimated glomerular filtration rate; FD, fast decline; MD, moderate decline; RS, relatively stable; MI, moderate increase; FI, fast increase.

**Table 3:** Demographic and clinical characteristics for the five different eGFR change groups.

Variables	FD	MD	RS	MI	FI	*P*
	(n = 821)	(n = 332)	(n = 390)	(n = 312)	(n = 744)	
Age ≥60 years, n (%)	382 (46.5)*	154 (46.4)*	210 (53.8)	156 (50.0)	377 (50.7)	0.112
Male, n (%)	494 (60.2)	184 (55.4)	236 (60.5)	186 (59.6)	414 (55.6)	0.242
Diabetes duration ≥10.0 years, n (%)	333 (40.6)	124 (37.3)	160 (41.0)	135 (43.3)	323 (43.4)	0.380
**Baseline characteristics**						
BMI ≥24.0 kg/m^2^, n (%)	543 (66.1)	198 (59.6)	243 (62.3)	200 (64.1)	443 (59.5)	0.063
BP <130/80 mmHg	270 (33.1)	117 (35.8)	133 (34.3)	100 (32.4)	270 (36.3)	0.616
LDL-C <2.60 mmol/L	322 (39.8)	142 (43.2)	163 (42.0)	144 (47.1)	337 (45.8)	0.095
Insulin use, n (%)	424 (51.6)*	157 (47.3)	176 (45.1)	147 (47.1)	352 (47.3)	0.211
SGLT-2i use, n (%)	25 (3.0)	15 (4.5)	9 (2.3)	5 (1.6)	15 (2.0)	0.106
GLP-1RA use, n (%)	10 (1.2)	5 (1.5)	2 (0.5)	5 (1.6)	9 (1.2)	0.689
ACEI/ARB use, n (%)	235 (28.6)	87 (26.2)	105 (26.9)	86 (27.6)	206 (27.7)	0.933
**Follow-up characteristics**						
BMI ≥24.0 kg/m^2^, n (%)	525 (63.9)	202 (60.8)	243 (62.3)	195 (62.5)	434 (58.3)	0.237
BP <130/80 mmHg	312 (38.2)	133 (40.5)	158 (40.6)	132 (42.6)	354 (47.6)*	0.005
LDL-C <2.60 mmol/L	377 (46.9)	155 (48.3)	176 (46.1)	160 (53.0)	355 (48.6)	0.406
Insulin use, n (%)	325 (39.6)	124 (37.3)	139 (35.6)	126 (40.4)	304 (40.9)	0.456
SGLT-2i use, n (%)	42 (5.1)	21 (6.3)	23 (5.9)	12 (3.8)	25 (3.4)*	0.138
GLP-1RA use, n (%)	14 (1.7)	7 (2.1)	3 (0.8)	5 (1.6)	9 (1.2)	0.563
ACEI/ARB use, n (%)	241 (29.4)	88 (26.5)	106 (27.2)	88 (28.2)	208 (28.0)	0.873

Data are numbers (%).

eGFR, estimated glomerular filtration rate; FD, fast decline; MD, moderate decline; RS, relatively stable; MI, moderate increase; FI, fast increase; *P < 0.05 compared with RS group; BMI, body mass index; BP, blood pressure; LDL-C, low-density lipoprotein cholesterol; SGLT-2i, sodium-glucose cotransporter-2 inhibitor; GLP-1RA, glucagon-like peptide-1 receptor agonist; ACEI, angiotensin-converting enzyme inhibitor; ARB, angiotensin receptor antagonist; UACR, urinary albumin-to-creatinine ratio.

### HbA_1c_ Reduction and eGFR Change

**Figure 2:**
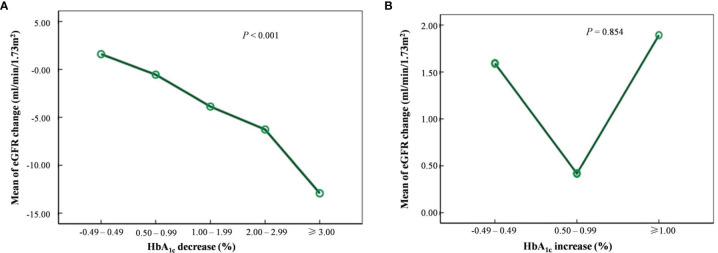
Trend of estimated glomerular filtration rate (eGFR) change in different HbA_1c_ decrease **(A)** and increase **(B)** groups.

A decreased trend in eGFR change was found among the HbA_1c_ decreasing groups (*P* < 0.001; **Figure 2A**) but not the HbA_1c_ increasing groups (**Figure 2B**). We evaluated the factors associated with the average eGFR changes. Overall, compared with an HbA_1c_ change of −0.49% to –0.49%, eGFR decreased significantly with an HbA_1c_ change of ≤−1.00%, even after multivariable adjustment (*P* < 0.001). Patients who showed an HbA_1c_ change of ≤−3.00% had the largest decline in eGFR (−12.61 ml/min per 1.73 m^2^) (**Table 4**). In the subgroup analysis (baseline HbA_1c_ <8.0% or ≥8.0%) of the association between HbA_1c_ reduction ranges and eGFR change, no significant association was found when baseline HbA_1c_ <8.0%; however, a significant association was found when baseline HbA_1c_ ≥8.0% (*P* < 0.001). The HbA_1c_ reduction played an important role in the eGFR decrease in subjects with hyperfiltration. The decline of eGFR reached −21.25 ml/min per 1.73 m^2^ when HbA_1c_ reduced ≥3.00%. This association was not found in the subgroup with no hyperfiltration (*P* = 0.076). When comparing groups of baseline UACR stages of A1 (<30 mg/g) and A2 (30–300 mg/g) and groups of diabetes duration of <10.0 and ≥10.0 years, a significant association was found between HbA_1c_ reduction range and eGFR change (*P* < 0.001). In general, eGFR FD was frequently found in patients with a baseline HbA_1c_ ≥8.0% and an HbA_1c_ change of ≤−3.00% or in patients with hyperfiltration and an HbA_1c_ change of ≤−1.00% and patients with UACR stage A2 and an HbA_1c_ change of ≤−3.00%. Regardless of a diabetes duration <10.0 or ≥10.0 years, eGFR FD was frequently found in patients with an HbA_1c_ change of ≤−3.00% (**Table 4**).

**Table 4:** Comparison of mean eGFR change in the different HbA_1c_ decrease groups.

Group	Subgroup	Number of patients	Multivariable adjusted eGFR change (95% CI)^#^	*P*
Total	HbA_1c_ change (%)	2,113		
	-0.49 to 0.49	934	1.32 (-0.24, 2.88)	<0.001
	-0.99 to -0.50	352	-0.73 (-2.95, 1.49)	
	-1.99 to -1.00	381	-3.18 (-5.35, -1.02)*	
	-2.99 to -2.00	187	-6.14 (-9.34, -2.94)*	
	≤-3.00	259	-12.61 (-15.74, -9.47)*	
Baseline HbA_1c_ (%)				
<8.0	HbA_1c_ change (%)	1,055		
	-0.49 to 0.49	719	1.33 (-0.12, 2.79)	0.114
	-0.99 to -0.50	217	-1.20 (-3.84, 1.44)	
	-1.99 to -1.00	107	-1.17 (-4.96, 2.63)	
	-2.99 to -2.00	12	9.50 (-1.70, 20.71)	
	≤-3.00	0		
≥8.0	HbA_1c_ change (%)	1,058		
	-0.49 to 0.49	215	-0.13 (-3.12, 2.87)	<0.001
	-0.99 to -0.50	135	-0.28 (-4.04, 3.48)	
	-1.99 to -1.00	274	-4.11 (-6.75, -1.47)	
	-2.99 to -2.00	175	-6.73 (-10.03, -3.43)*	
	≤-3.00	259	-11.43 (-14.21, -8.65)*	
Baseline hyperfiltration				
Yes	HbA_1c_ change (%)	1,181		
	-0.49 to 0.49	448	-4.61 (-7.05, -2.18)	<0.001
	-0.99 to -0.50	179	-7.04 (-10.38, -3.70)	
	-1.99 to -1.00	242	-10.61 (-13.47, -7.74)	
	-2.99 to -2.00	125	-15.76 (-19.91, -11.62)*	
	≤-3.00	187	-21.25 (-25.17, -17.33)*	
No	HbA_1c_ change (%)	932		
	-0.49 to 0.49	486	9.11 (7.23, 10.99)	0.076
	-0.99 to -0.50	173	7.17 (4.33, 10.01)	
	-1.99 to -1.00	139	7.06 (3.76, 10.35)	
	-2.99 to -2.00	62	8.13 (3.08, 13.17)	
	≤-3.00	72	0.63 (-4.63, 5.88)	
Baseline UACR (mg/g)				
<30	HbA_1c_ change (%)	1,609		
	-0.49 to 0.49	764	1.05 (-0.59, 2.69)	<0.001
	-0.99 to -0.50	263	-1.36 (-3.93, 1.21)	
	-1.99 to -1.00	281	-3.05 (-5.61, -0.50)*	
	-2.99 to -2.00	131	-3.36 (-7.18, 0.47)*	
	≤-3.00	170	-9.59 (-13.13, -6.06)*	
30–300	HbA_1c_ change (%)	504		
	-0.49 to 0.49	170	-1.40 (-4.95, 2.16)	<0.001
	-0.99 to -0.50	89	-0.14 (-4.59, 4.31)	
	-1.99 to -1.00	100	-1.95 (-6.18, 2.29)	
	-2.99 to -2.00	56	-9.48 (-15.25, -3.70)	
	≤-3.00	89	-13.32 (-18.13, -8.50)*	
Diabetes duration (years)				
<10.0	HbA_1c_ change	1,256		
	-0.49 to 0.49	556	1.36 (-0.74, 3.46)	<0.001
	-0.99 to -0.50	198	-0.16 (-3.17, 2.85)	
	-1.99 to -1.00	215	-2.97 (-5.87, -0.06)*	
	-2.99 to -2.00	103	-8.96 (-13.34, -4.58)*	
	≤-3.00	184	-13.09 (-17.08, -9.09)*	
≥10.0	HbA_1c_ change	857		
	-0.49 to 0.49	378	1.43 (-0.92, 3.78)	0.001
	-0.99 to -0.50	154	-1.35 (-4.65, 1.96)	
	-1.99 to -1.00	166	-3.36 (-6.63, -0.08)	
	-2.99 to -2.00	84	-2.96 (-7.71, 1.78)	
	≤-3.00	75	-12.38 (-17.64, -7.12)*	

eGFR, estimated glomerular filtration rate; CI, confidence interval; UACR, urinary albumin-to-creatinine ratio.

*P < 0.05 compared with HbA1c change of -0.49 to 0.49% (checked with Bonferroni).

^#^Adjusted for category of age, gender, disease duration, baseline hyperfiltration, BMI, HbA_1c_, UACR stage, and follow-up BP control, insulin, SGLT-2i, and ACEI/ARB usage.

## Discussion

The eGFR is a marker used to evaluate renal function and to predict the risk for ESRD and renal death in diabetes cases, whereas the implication of creatinine-based eGFR is limited to hyperglycemia status. Due to hyperglycemia-related hyperfiltration, the measured GFR decreased from 149 to 129 ml/min per 1.73 m^2^ (16% reduction) after HbA_1c_ dropped from 10% to 7% and increased significantly toward the baseline values along with HbA_1c_ control deterioration in patients with T1D (20). Additionally, HbA_1c_ was positively associated with eGFR, whether independently or together with fasting plasma glucose (FPG), in participants with prediabetes and was associated with significantly increased odds ratios (ORs) of hyperfiltration (21, 22). It was reported that every 1% increase in HbA_1c_ levels and a 2.0 mmol/L increase in 2-h post-load glucose (2hPG) were associated with a 17% and 61.4% higher risk of hyperfiltration, respectively (23, 24). It was also demonstrated that eGFR equations were less accurate in the diabetic group than in the non-diabetic group, and HbA_1c_ was an independent factor associated with the accuracy of eGFR equations (12). This study aimed to identify the association between short-term (≤12 months) changes in eGFR and HbA_1c_ in patients with T2D and to provide perspectives for carefully interpreting eGFR changes in clinical practice.

Several patients with T2D experienced eGFR FD or MD during the median 8-month follow-up. From group FI to FD, baseline HbA_1c_ level, HbA_1c_ reduction range, and the age-adjusted prevalence of hyperfiltration increased gradually. Patients with FD had the highest baseline HbA_1c_ level, HbA_1c_ reduction, and a prevalence of hyperfiltration as high as 78.4%. A downward trend of eGFR change was demonstrated along with HbA_1c_ reduction, even in subgroup analysis after multivariable adjustment, regardless of its value at stages A1 and A2 or with a diabetes duration <10.0 or ≥10.0 years, indicating a close association between eGFR decline and HbA_1c_ reduction. This may be due to the HbA_1c_ reduction itself, but not as a result of hyperfiltration, since the association remained significant even after adjusting for the presence of hyperfiltration. This was not the case at first sight. Existing research demonstrated HbA_1c_ reduction after a few months to be associated with a significant reduction in eGFR, and the correlation could be extended to both types of diabetes and more advanced stages of renal impairment. Moreover, each +1% of HbA_1c_ was associated with +5.3 ml/min per 1.73 m^2^ eGFR using the MDRD equation (14). In day-to-day clinical practice, the prediction of GFR becomes crucial when renal function declines. We note here that eGFR and its estimations would be significantly higher in poorly controlled patients. The reduction of HbA_1c_ by ≥3.00% will possibly lead to a fast decline in eGFR of ≥10.0% ml/min per 1.73 m^2^ and reflect true renal function.

There may be concerns about whether a decrease in eGFR indicates the decline of renal function, since the link between acute intensive glycemic control and acute neuropathies or DR progression is described in the literature (25–28). The neuropathies experienced by patients were acute, severe but reversible, and did not occur as a consequence of chronic hyperglycemia. The most important factors for the early worsening of DR were a higher HbA_1c_ level at screening and a reduction in this level during the first 6 months of treatment (28). However, we cannot deny the long-term benefits of blood glucose control. The retinal morphology improved during the following years, and intensive glycemic control continued to reduce DR progression (25, 27, 29). Early worsening of diabetic nephropathy was found in T2D after rapid improvement in chronic severe hyperglycemia, with eGFR falling by 23–35 ml/min per 1.73 m^2^ in the first year and the most significant loss occurring in the first 6 months (mean 41 ml/min/year), however, followed by a slower rate of loss (30). A transient decrease in eGFR during the intervention period and its return to near the baseline level at 104 weeks with SGLT-2i treatment indicated possible similar pathophysiological mechanisms and the development of a process for HbA_1c_ reduction (31, 32). A threshold for HbA_1c_ levels related to the risk of complications was observed. Above the threshold of 6.5%, every 1% increase in HbA_1c_ level was associated with a 40% higher risk of a microvascular event, including CKD (33). No significant downward trend in eGFR changes was found in patients with a baseline HbA_1c <_8.0%, indicating a threshold for suboptimal glycemic control but not an increased risk for rapid eGFR decline.

The largest eGFR decline trend, along with HbA_1c_ reduction, was found in patients with hyperfiltration. The age-adjusted prevalence of hyperfiltration in this study was higher than that in a previous report (34). The reason for this difference may have resulted from the different GFR measurement methods, definition criteria, study population, and HbA_1c_ levels (19, 34). It was found that a more considerable reduction in eGFR at 6 months significantly predicted a slower subsequent decline, and the amelioration of hyperfiltration was significantly associated with a slower long-term eGFR decline on follow-up (19). Patients with hyperfiltration may benefit from an eGFR decline to the normal range. It is noted that hyperfiltration was also observed in subjects with a diabetes duration of ≥9.0 years and an HbA_1c_ level of approximately 8.3% (34). Thus, caution is advised when interpreting eGFR before reaching adequate glycemic control and the amelioration of hyperfiltration, even in patients with long-term diabetes duration. Moreover, since eGFR equations were considered less accurate in patients with diabetes, regardless of using either the CKD-EPI or MDRD equations, eGFR changes and their response to treatment should be monitored better and regularly alongside HbA_1c_.

This study identified the association between short-term changes in eGFR and HbA_1c_ in patients with T2D during the 12-month follow-up period. Some patients with T2D experienced eGFR FD or MD during the median 8-month follow-up period. Since a downward trend in eGFR change was demonstrated alongside an HbA_1c_ reduction, regardless of the UACR stage and diabetes duration independent of hyperfiltration, sustained monitoring and the cautious interpretation of HbA_1c_ and eGFR changes are required in clinical practice. We do not know whether the rapid eGFR decline associated with an HbA_1c_ reduction in this analysis will partly recover in future follow-ups, which requires further observation.

## Limitations and Strengths

A weakness of this study concerns its retrospective nature. Thus, the study findings are hypothesis-generated and require further testing. The major strengths of the study were that it included a large study population and demonstrated an association between HbA_1c_ changes and short-term eGFR changes in populations with T2D; furthermore, all subjects were prospectively monitored using gold-standard procedures. The results may thus present extensive external validity.

## Data Availability Statement

The original contributions presented in the study are included in the article/supplementary material. Further inquiries can be directed to the corresponding author.

## Ethics Statement

The studies involving human participants were reviewed and approved by the Ethics Committee of Beijing Ruijing Diabetes Hospital. Written informed consent for participation was not required for this study in accordance with the national legislation and the institutional requirements.

## Author Contributions

LA and JL conceived and designed the study. LA analyzed the data and drafted the article. JL and YLe reviewed/edited the article. All authors contributed to data collection, critically reviewed the article, and approved the final version to be published.

## Funding

This work was supported by the Beijing Fengtai District health system project (No. 2017-81).

## Conflict of Interest

The authors declare that the research was conducted in the absence of any commercial or financial relationships that could be construed as a potential conflict of interest.

## Publisher’s Note

All claims expressed in this article are solely those of the authors and do not necessarily represent those of their affiliated organizations, or those of the publisher, the editors and the reviewers. Any product that may be evaluated in this article, or claim that may be made by its manufacturer, is not guaranteed or endorsed by the publisher.
